# Demographic drivers of Norway rat populations from urban slums in Brazil

**DOI:** 10.1007/s11252-020-01075-2

**Published:** 2020-12-18

**Authors:** C. G. Zeppelini, T. Carvalho-Pereira, R. Sady Alves, D. C. C. Santiago, V. F. Espirito Santo, M. Begon, F. Costa, Hussein Khalil

**Affiliations:** 1 Laboratório de Mamíferos, Universidade Federal da Paraíba, Cidade Universitária, João Pessoa, Brazil; 2 Programa de Pós-Graduação em Ecologia: Teoria, Aplicações e Valores, Universidade Federal da Bahia, R. Barão do Geremoabo, Salvador, 147 Brazil; 3 Instituto de Saúde Coletiva, Universidade Federal da Bahia, R. Basílio da Gama, S/N, Salvador, Brazil; 4 Programa de Pós-Graduação em Ciência Animal nos Trópicos, Universidade federal da Bahia, R. Barão do Geremoabo, Salvador, 147 Brazil; 5 Instituto de Biologia, Universidade Federal da Bahia, R. Barão do Geremoabo, Salvador, 147 Brazil; 6 Faculdade de Farmácia, Universidade Federal da Bahia, R. Barão do Geremoabo, Salvador, 147 Brazil; 7 Institute of Integrative Biology, University of Liverpool, Biosciences Building, Liverpool, L69 7ZB UK; 8 Department of Wildlife, Fish and Environmental Studies, Swedish University of Agricultural Sciences, SE-901 83 Umeå, Sweden

**Keywords:** Rattus norvegicus, Urban ecology, Demography, Tropics, Slums, Zoonotic disease, Salvador

## Abstract

The Norway rat is a globally distributed pest, known for its resilience to eradication and control programs. Efficient population control, especially in urban settings, is dependent on knowledge of rat demography and population ecology. We analyzed the relationship between four demographic outcomes, estimated by live-trapping data, and fine-scale environmental features measured at the capture site. Wounds, a proxy for agonistic interactions, were associated with mature individuals. Areas with environmental features favorable to rats, such as open sewers and unpaved earth, were associated with more mature individuals with a better body condition index. The control measures (environmental stressors) are likely to be disrupting the social structure of rat colonies, increasing the frequency and distribution of agonistic interactions, which were common in both sexes and maturity states. The relationship between the favorable environmental conditions and the demographic markers analyzed indicate possible targets for infestation control through environmental manipulation, and could be incorporated into current pest management programs to achieve long-term success. Our study indicate that urban interventions focused on removal of potential resources for rats could be potential long-term solutions by reducing the carrying capacity of the environment.

**Supplementary Information:**

The online version contains supplementary material available at 10.1007/s11252-020-01075-2.

## Introduction

The Norway rat (*Rattus norvegicus*) is one of the most successful worldwide colonizers in evolutionary history (Morand et al. 2015). The genus *Rattus* has historically tracked human movement, dispersing from its origins in Asia to Europe, and then to the rest of the world by the advent of long-distance routes such as the Silk Route and the trans-Atlantic navigations (Puckett et al. 2016). Today, the Norway rat is present in all populated continents and its occurrence is closely associated with humans (Himsworth et al. 2014), being able to colonize a diversity of man-made habitats (urban areas, parklands, farmlands) (Glass et al. 1989).

Its adaptability to a variety of human-created habitats, particularly urban areas (McKinney 2002), and their biological traits, make the Norway rat a ubiquitous pest. In agricultural areas they damage crops and contaminate stored harvests, and in urban environments they cause structural damage (e.g. gnawing on electric wires, damaging house structures by burrowing) and are reservoirs to several zoonotic pathogens (Desvars-Larrive et al. 2017). The estimated damage caused by rats reaches hundreds of billions of dollars annually, greater than the calculated damage caused by, for example, air pollution mortality (Parsons et al. 2017).

In urban settings, rat infestation is usually associated with poor communities (slums, sensu Costa et al. (2014a)), where precarious infrastructure and deficient urban services such as trash collection and adequate sanitation create favorable conditions for high rat population density (Minter et al. 2018). In slums, rats carry and transmit both bacterial and viral pathogens (Costa et al. 2014a). Amongst the diversity of pathogens associated with rat infestations, leptospirosis is the greatest public health threat, with especially high incidence in urban areas and slums, where the environment facilitates disease transmission (Costa et al. 2014a; Garchitorena et al. 2017; Himsworth et al. 2013).

Aside from a few successes on islands (Innes 2001; Robertson and Gemmell, 2004), rats have resisted most eradication efforts. Control has relied heavily on rodenticide application (Costa et al. 2014b), but populations usually recover within a few months (Masi et al. 2009). This method also involves risks to non-target species (Santos et al. 2017), and resistance to rodenticides can develop over time (Lutermann et al. 2017). It is thus necessary to transition to a strategy focused on managing the landscape and the resources exploited by rats (Colvin et al. 1996). However, the design of effective control measures depends on a solid understanding of the ecology of the target species in general and especially at the site to be managed. This is necessary to inform decisions regarding management options, such as defining eradication units (i.e., the spatial unit for management), timing of implementation, and selecting adequate interventions (Parsons et al. 2017; Robertson and Gemmell, 2004).

Despite the proximity between humans and rats, our knowledge of rat ecology in urban settings is still incipient (Combs et al. 2018). Most studies have been conducted in developed areas with temperate climate, with an emphasis on rats’ use of space and movement (Byers et al. 2017; Davis and Fales, 1950; Davis and Hall 1951; Himsworth et al. 2015; Himsworth et al. 2014), which have limited relevance to the less organized reality of developing countries in the tropics (Desvars-Larrive et al. 2018). Moreover, little is known about how the environment influences rats’ demography on a local scale.

One of the main challenges faced today in pest control is understanding the demography and ecology of the species of interest (Cavia et al., 2009; Dexter 2003; Edwards et al. 2004; Hampton et al. 2004; Zenger et al. 2003), which may define the spatial and temporal parameters of an intervention taking in account reproduction, variations in spatial occupation and temporal phenomena. Furthermore, in scenarios where the target species involved is a zoonotic reservoir, population structure is relevant for current and future migration (as the use of space, home range and migration differ between sexes and maturity stages), and to the rate of transmission among infected and newly recruited or immigrant individuals (Byers et al. 2019b). The present study aims to describe demographic data for urban *Rattus norvegicus* populations at four study sites in Salvador, Brazil, to investigate the role of environmental factors and control efforts in determining the population structure in these sites. Our hypotheses are that resources (e.g. trash, water bodies) and other favorable environmental traits (e.g. burrowable soil) will foster a more stable rat population with better body condition, whereas the deployment of rodent control efforts will have negative effects on the populations (lower body condition, survival and more aggression).

## Materials and methods

### Study areas

**Fig. 1:**
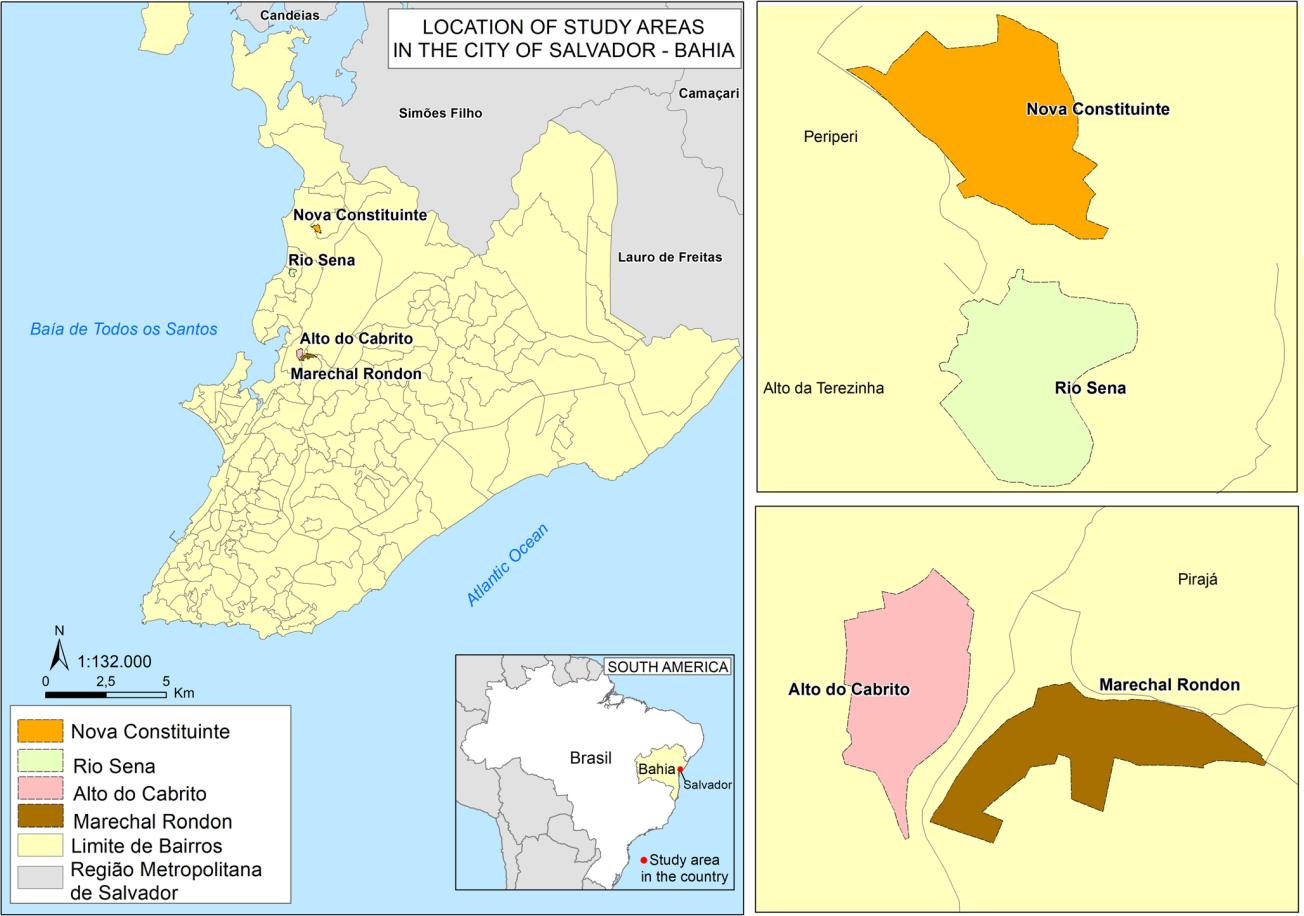
Map of the four communities studied in the city of Salvador, Bahia, Brazil. MR = Marechal Rondon; AC = Alto do Cabrito; RS = Rio Sena; NC = Nova Constituinte. Map produced by Dr. Ricardo Lustosa Brito

Four urban slum communities (sensu Costa et al. (2014a)) were sampled in the city of Salvador, Bahia in Brazil, namely Marechal Rondon (MR), Alto do Cabrito (AC), Rio Sena (RS) and Nova Constituinte (NC) (Fig. 1). We live-trapped rats during two campaigns in each community. The first campaign was between April and June 2018, and the second between October and November 2018, representing rainy and dry seasons, respectively. The sampling areas were between 0.07 and 0.09 km^2^ with an altitudinal gradient (slopes and valley bottoms) characteristic of urban slums in the city of Salvador. The areas were selected by the similarity of their conditions (socioeconomic, geographic relief, house and urban infrastructure) and their location within the same sanitary district in the Zoonosis Control Center classification, characterized by high numbers of complaints about rat infestation, as well as leptospirosis cases records.

### Sampling and data collection

Sampling locations were selected using conditioned random sampling. Every residence in the study area received a number and a geotag within the area, and forty evenly spaced random points were selected for sampling, in order to adequately represent the community. Two Tomahawk live traps baited with sausage were deployed in each point for four nights, totaling 320 attempted trap-nights per campaign in each area and 640 trap-nights per community. The traps were located in peri-domestic settings such as back yards after obtaining permission from the residents.

For each sampling point, an environmental survey questionnaire was applied by field personnel through visual inspection of the peri-domestic environment and interviewing the head of the household. We gathered data on environmental and socioeconomic variables, such as the presence of trash accumulation sites and visits from the Zoonosis Control Center (CCZ) agents (Table 1, Supplementary Material 1), which were potential explanatory variables for the demographic features of rats within the communities.

**Table 1:** Variables collected for the models based in the Integrated Pest Management outdoor inspection form (CDC) for environmental factors associated to rodent infestation, as recorded within the 20-m buffer around each sampled household

Variable	explanation and formatting
Type of ground coverage	Categorical (pervious, semi-pervious, impervious). Whether the soil in the buffer is paved or not
Presence of sewers	Presence/absence. Presence of a sewer canal within the 20 m buffer of the sampling point (household)
Water bodies	Categorical (water body, leak, puddle, absent). Presence and type of water body or other water source
Available residues	Presence/absence. Presence of construction debris and/or household trash
Opossums	Presence/absence. Whether there were opossums captured in the sampling point/buffer
Dogs	Presence/absence and counts. Whether there are dogs residing within the buffer zone (household reported)
Cats	See dogs
CCZ visits	Presence/absence. Whether the Zoonosis Control Center (CCZ) has visited the area in the last 12 months (household reported)
Use of rodenticides	Presence/absence. Whether the local residents deploy some type of chemical rodenticide in the area in the last 12 months (household reported)
Presence of Capillaria hepatica	Presence/absence. Whether the necropsied individual presented signs of *C. hepatica* in the liver.

After capture, rats were transported to a field lab, where they were euthanized according to a standardized protocol (sedation induced by Isoflurane, followed by euthanasia by intraperitoneal sodium thiopental) (Costa et al. 2015). The animals were then taxonomically identified, body measures taken, and dissected for the collection of biological samples. During the dissection of the specimen, the head, paws and tail were inspected for wounds or scars. Wounds were analyzed only in terms of presence or absence as a proxy for agonistic interactions and stress. In examining specimens, it was not possible to determine whether the wound intensity observed is the outcome of one or few intense attritions, or several lower intensity encounters. During dissection, the presence of *Capillaria hepatica* infestation was observed by examining the liver for the presence of fibrosis scars visible on the surface of the organ (Stojčević et al. 2002) as a possible co-measure for health. For the body condition index, we used the Scaled Mass Index (SMI), which is based on the weight-length ratio, whilst accounting for the effect of age (Peig and Green, 2009), allowing to use body size as a proxy for health. Pregnant individuals had their weight adjusted following the method by Minter et al. (2017). Animals were classified as mature if a) they were over the 200 g threshold, considered as a viable surrogate for sexual maturity (Porter et al. 2015) or b) they presented physical sexual maturity signs (i.e. fully descended scrotal testes for males, perforated vagina for females). All procedures were performed under ethical approval by the Ethical Committee of the Animal Use (CEUA) protocol number 019/2016 of the IGM – Oswaldo Cruz Foundation (Fiocruz).

### Data analysis

To investigate the main environmental drivers for demographic parameters, namely the sex ratio (proportion of males to females), maturity ratio (proportion of adults to juveniles), presence of wounds and SMI, we used Generalized Linear Mixed Effect Models (glm). As explanatory variables, we used the covariates informed from the questionnaires and field data (Supplementary Material 1). We used area (each of the 4 communities) as a fixed effect.

For each outcome, we defined a pool of potential explanatory variables (Table 1) based on our hypotheses (Supplementary Material 1 presents the variables and their hypothesized effect on parameter of interest). To check for collinearity among the explanatory variables, we used Pearson’s chi-squared or Fisher’s Exact test for categorical data, or a Spearman’s Rank Sum Correlation for count data. The covariate pool for the sex model was formed by ten variables (presence of opossums, available residues, category of water body, presence of sewers in a 20-m radius, CCZ (Zoonosis Control Center) visits, application of rodenticide poison baits by the population, presence and number of cats, presence and number of dogs). There were11 covariates for the maturity model (sex, presence of opossums, available residues, category of water body, presence of sewers in a 20-m radius, CCZ visits, application of rodenticide poison baits, presence and number of cats, presence and number of dogs, type of ground coverage); 15 for wounds (sex, presence of opossums, available residues, category of water body, presence of sewers in a 20-m radius, CCZ visits, application of rodenticide poison baits, presence and number of cats, presence and number of dogs, type of ground coverage, maturity, SMI, accumulated materials). For SMI, there were 14 covariates (sex, presence of opossums, available residues, category of water body, presence of sewers in a 20-m radius, CCZ visits, application of rodenticide poison baits, presence and number of cats, presence and number of dogs, type of ground coverage, maturity, accumulated materials).

Before fitting the full model, for presence of wounds, we ran a primary model including sex, maturity and the interaction between the two. With the defined covariate pool for each outcome, we created a multivariable global model, and ran a model selection using Akaike’s Information Criterion (AIC). The models discussed are the ones considered plausible (ΔAIC <2 compared to the model with the lowest AIC) (Burnham and Anderson 2002). We chose as the most parsimonious model, the one with a ΔAIC <2 and the fewest number of explanatory variables. To determine the strength of support for the explanatory variables considered in the set of models, the sum of ‘Akaike weights’ over all models including the explanatory variable (or simply, ‘relative variable importance’) was used. All analyses were performed in R v3.5.1 using the packages ‘Tidyverse’, ‘lme4’ and ‘MuMIn’ (Barton 2018; Bates et al. 2015; R Core Team 2018; Wickham 2017).

## Results

### Descriptive statistical summary

In the two campaigns, we captured a total of 118 individuals of *Rattus norvegicus* (trap success of 0.2063 captures per trap-night), of which three were removed from the analysis due to absence of sex or maturity data (Table 2). We lost fourteen trap-nights in Alto do Cabrito due to flooding, as well as 17 in Marechal Rondon, 15 in Rio Sena and 22 in Nova Constituinte. The majority of captures were adults (*N* = 85), with a sex ratio slightly skewed towards females at 1:1.21. For both sexes, adults had more wounds. Notably, all adult females showed wounds or scars. Wounds were frequent in adults (80/85) and juveniles (23/30).

**Table 2:** Demographic summary of the captured *Rattus norvegicus* in four communities, means with S.D. giver in parenthesis. Females have two values for weight due to the presence of pregnant females. Values in bold font have been corrected to account to pregnant individuals

		Body Mass (g)	Body Lenght (mm)	SMI	Wounds		Presence of *Capillaria hepatica* scars in liver
					present	absent	
Male	adult (*N* = 46)	274.83 ± 86.53 [105–460]	211.65 ± 19.51 [163–250]	275.42 ± 38.6 [195.23–370.46]	41	5	30
	sub-adult (*N* = 6)	90.42 ± 59.9 [30–195]	142.17 ± 26.51 [105–179]	279.22 ± 52.7 [183.67–338.12]	3	3	1
Female	adult (*N* = 39)	302.63 ± 63.06 **(261.4 ± 93.28)** [145–400] **[30–460]**	221.46 ± 18.48 [173–273]	276.49 ± 41.87 [164.69–380.58]	39	0	32
	sub-adult (*N* = 24)	213 ± 79.46 [85–350]	198.67 ± 27.42 [150–237]	257.62 ± 42.35 [196.86–349.99]	20	4	15
Total	adult (N = 85)	291.44 ± 82.33 [105–460]	216.15 ± 19.56 [163–273]	271.78 ± 40.09 [164.69–380.58]	80	5	62
	sub-adult (*N* = 30)	188.48 ± 90.07 [30–350]	187.37 ± 35.29 [105–237]	261.94 ± 44.48 [183.67–349.99]	23	7	16

### Outcome models

#### Sex

The model selection recovered the null model (sex ratio ~ 1) as the most parsimonious of the 17 models with ΔAIC<2.

#### Maturity

For the 18 plausible models (ΔAIC<2) in the classification, two variables (presence of sewers and sex) were present in all models; while type of water body was present in all but two of the plausible models (Supplementary Material 2). The most parsimonious model included presence of sewers and sex, (ΔAIC = 0.84). The model considered being male (OR 5.597, *p* = 0.001) and the presence of sewers (OR 3.336, *p* = 0.01) as positively associated with the likelihood of capturing adults (Table 3).

**Table 3:** Size and effect of the variables that compose the selected models, with lower (2.5%) and upper (97.5%) confidence intervals and statistical significance

	OR	2.5%	97.5%	Std. Error	*P* value
Maturity					
(Intercept)	0.766	0.351	1.625	0.3868	0.489
Presence of sewers	3.336	1.344	8.667	0.4722	0.010
Sex (male)	5.597	2.104	17.159	0.5286	0.001
Wounds: model 1					
(Intercept)	8.787	2.523	57.655	0.7138	0.002
Presence of dogs	1.759	1.084	3.905	0.3023	0.061
Maturity (adult)	23.793	3.938	213.606	0.9955	0.001
Sex (male)	0.075	0.008	0.444	1.0056	0.01
Presence of rodenticide	0.122	0.022	0.544	0.8013	0.008
Wounds: model 2					
(Intercept)	7.485	2.115	37.016	0.7148	0.004
CCZ activity	7.623	1.447	72.908	0.9603	0.003
Maturity (adult)	20.516	3.442	197.763	1.0033	0.002
Sex (male)	0.064	0.006	0.405	1.0632	0.009
Presence of rodenticide	0.206	0.044	0.839	0.7350	0.031
	*β*	*2.5%*	*97.5%*	*Std. Error*	*P value*
SMI					
(Intercept)	272.99	249.033	296.947	12.223	< 2e-16
CCZ activity	−24.007	−39.195	−8.818	7.749	0.002
Accumulated materials	−23.725	−46.192	−1.258	11.463	0.040
Presence of sewers	18.426	3.297	33.554	7.719	0.018
Unpaved soil	23.208	6.833	39.583	8.355	0.006

The relative variable importance assigned the strongest support for sex (weight = 0.99) and presence of sewers (0.95) all present in 4096 models.

#### Wounds

The variables “presence of cats” and “number of cats” were responsible for causing a “perfect separation” error during the model run, and so “number of cats” was dropped from the model.

There were 24 models within the ΔAIC threshold. Two parsimonious models were selected, one comprising presence of dogs, maturity, sex and rodenticides, while the second substituted presence of dogs for CCZ activity (Supplementary Material 2). Both models detected a large negative effect of the presence of rodenticide (model 1: OR 0.122, *p* = 0.008; model 2 OR 0.206, *p* = 0.03), and being male (model 1 OR 0.075, *p* = 0.01; model 2 OR 0.06, *p* = 0.009), as well as a strong positive effect of being a sexually mature individual (model 1 OR 23.793, p = 0.001; model 2 OR 20.516, *p* = 0.002) in the chance of capturing wounded animals. The second parsimonious model, however, also detected the effect CCZ activity in the area (OR 7.715, *p* = 0.034) (Table 3).

The relative variable importance assigned the strongest support for maturity (weight = 0.99), sex (0.96), the use of rodenticide poison (0.9), number of dogs (0.71), and CCZ activity (0.58), all present in 8192 models.

#### SMI

The model selection recovered 15 models within the ΔAIC threshold. The most parsimonious model included CCZ activity, accumulated materials, presence of sewers and type of soil coverage (Supplementary Material 2), which were present in all 15 models, and were all considered as having a significant effect on SMI. CCZ activity (β −24.0, p = 0.002) and accumulated materials (β −23.7, *p* = 0.04) had a negative effect, while presence of sewers (β 18.4, p = 0.01) and type of ground coverage (β 23.2, *p* = 0.006) had a positive association (Table 3).

The relative variable importance assigned the strongest support for CCZ activity (weight = 0.87), accumulated materials (0.77), presence of sewers (0.75), type of ground coverage (0.72), sex (0.54) and number of dogs (0.54), all present in 16,384 models.

## Discussion

Here, we observed that environmental features related to resources or shelter, such as open sewers, could affect the characteristics of urban Norway rat populations. On the other hand, environmental stressors (CCZ visits and rodenticides, which remove individuals from the population and could be considered an environmental pressure) decreased SMI.

Wounds and scars, used as a proxy for agonistic interactions (Feng and Himsworth, 2014), were present mostly in adults (Glass et al. 1989; Himsworth et al. 2014). However, the association between wounds and being female in our model – wounds were 9% more frequent in females, and all adult females were wounded – contradicted previous association between wound prevalence and being male (Himsworth et al. 2014), as well as another study that found no association between sex and wounding (Costa et al. 2014a). Agonistic encounters may be driven by sex-specific factors that could lead to fights over social hierarchy and territory. Male and female adults display aggressive behaviors towards intruders, although female aggression tends less frequently to wound the invader, whereas males often retaliate and wound (Blanchard et al. 1984). Female aggression could also be linked to parental defense, as postpartum and lactating females in particular display aggressive behaviors towards intruders and other potential threats (Gioverdani et al. 2000).

In either scenario, the frequency of aggression could be exacerbated by environmental disturbances (in this case, the control efforts), which may cause the concentration of individuals into favorable (and/or disturbance-free) areas where colonies and/or parous female nests are located, increasing the density there and thus the chances of agonistic encounters. Whether the presence of the control measures does lead to the spatial concentration of individuals needs to be tested. There is currently a paucity of studies on wild rat behavior (Clapperton 2006; Macdonald et al. 1999), hindering any association between the effect detected and potential behavioral causes. The effect of maturity on wounding is expected due to the longer lifespan offering more opportunities to incur in aggressive encounters, as well as the role of sexually mature individuals in territorial defense.

Rodent control methods, which were expected to act as a disturbance and thus expected to cause more wounding, greatly diminished the capture of wounded rodents. Our assumption was based on experimental studies which found that manipulation of socio-environmental factors in rat colonies can cause changes in aggressiveness, especially when territory is manipulated (Meaney and Stewart, 1979). The negative association found in our study could indicate that there is a trappability bias in areas where constant rodenticide application and CCZ visits occur, removing the older, more experienced individuals (and likely more wounded), increasing the odds of more naïve adults being captured. This could be one of the environmental features that regulate rat trappability in urban settings (Byers et al. 2019a).

SMI was affected by a range of factors, including sex and maturity, both of which relationships have been detected previously (Panti-May et al. 2016). The application of rodenticides by the CCZ negatively influenced the SMI, while resources such as sewers and exposed earth increased the SMI in accordance with our hypotheses. ‘Accumulated materials’, however, showed a negative association with the SMI. This may indicate that there is a need for a finer separation of the variables to understand the ecological drivers of body condition, as this variable includes both construction debris and inorganic household refuse (such as broken home appliances, boxes, etc.), which could have different utility as hiding places, pose threats to animal occupation (i.e. presence of broken glass, sharp objects) and interact differently with the surrounding environment. It is also likely that accumulated materials diminish environmental quality, which reflects in the body condition of the occupant individuals.

The majority of individuals in the four study areas were adults, which is different from what was reported in other areas in Brazil (Panti-May et al. 2016), but has been detected in other studies in the Americas and Europe in an urban context (e.g. Desvars-Larrive et al. (2017); Himsworth et al. (2014); Villafañe et al. (2013)). Two out of four variables selected by the models (sewers, water bodies) are resources, which could explain the higher occurrence of adults, as resource availability is one of the main factors dictating home range selection on rats, with resource-abundant areas being normally held by adult individuals (Byers et al. 2019b; Macdonald et al. 1999), especially as water puddles are more likely to be used as drinking sources, in contrast to our earlier hypothesis. The effect of sex is likely present due to 88.4% of the males being adult.

With a sex ratio close to one to one (54.7% females), the populations studied differ from what has been registered in higher latitudes (Desvars-Larrive et al. 2017; Glass et al. 1989; Himsworth et al. 2014), while other studies performed in Brazil have recorded similar sex ratios (Panti-May et al. 2016; Porter et al. 2015), possibly indicating a pattern for populations in tropical urban areas. A population dominated numerically by females, however, seems to be the exception (e.g. Villafañe et al. (2013)). The higher number of females could also indicate a population response to the abundance of food and mild climate throughout the year, as populations in the tropics display continuous reproduction due to a year-round warm weather (Panti-May et al. 2016), but this can only be verified with a long-term study.

Our main findings give empirical support to the need for the transition into a landscape management practice (Colvin et al. 1996). Within these findings, we confirm the importance of managing sewers and water bodies, important resources and pathways for Norway rat movement, within areas with rat infestation (Gurtler et al. 2008; Masi and Pino 2011; Traweger et al. 2006), so naturally efforts in closing sewers and improving the sanitation around water bodies could have a positive effect in reducing rat infestation by reducing the available water supply for rat colonies, while also protecting the local population from contamination from zoonosis such as leptospirosis (Felzemburgh et al. 2014), which are linked both to contaminated water and rat infestation. We have also found that the presence of burrowable, unpaved ground is a key resource for the presence of rats in good health condition within an area, which points to two pathways to manage the landscape: i) pavement and maintenance of streets and paths, ii) management and maintenance of vacant lots and yards to avoid their use as burrow sites. With an organized pavement, drainage and landscaping plan, it is possible to greatly reduce the environmental carrying capacity (Lambert et al. 2017; Orgain and Schein 1953) of the areas and impede the continuous poverty-trap of urban neglect and pest infestation.

## Supplementary Information

ESM 1 (XLSX 11 kb)

ESM 2 (DOCX 21 kb)

## Data Availability

The data used in this study is stored in a RedCap database at Instituto Gonçalo Moniz – FIOCRUZ/BA, currently accessible for personnel only.
