# Supplementary material for: Demographic drivers of Norway rat populations from urban slums in Brazil
Source: Urban Ecosyst. 2020 Dec 18;24(4):801–9. doi: 10.1007/s11252-020-01075-2 (PMC8550123; doi:10.1007/s11252-020-01075-2)
Supplement: Supplementary file 2 — (DOCX 21 kb) [file 11252_2020_1075_MOESM2_ESM.docx]

Supplementary Material.

Section 1 – Hypothesis for variable selection.

Each variable can be classified either as a “resource”, being any kind of resource or environmental feature that in one or other state facilitates or is fundamental to the presence of rats in the area as measured by captures; or as a “stressor”, any kind of negative impacting feature that can diminish the probability of having rats occupying the area. Sex and maturity have been added to all models as to account for the possibility of sex and age-specific variations in behavior (adult and male Norway rats have larger home range, explore and disperse more, and are more aggressive, thus more likely to face situations such as finding poison baits and entering households). SMI will be added to the wounds model as dominant and aggressive individuals tend to be larger; age and body size/condition indicate whether an individual is a possible dominant (and prone to be aggressive towards younger, smaller, more naïve individuals).

Resources

- Type of ground coverage (presence-absence)
- Presence of sewers within the buffer (presence-absence)
- Presence and type of water bodies (categories: water body, puddle, leakage, absent)
- Available residues [both domestic trash and debris from construction work] (presence-absence)

Stressors

- Capture of opossums (presence-absence)
- Dogs (presence-absence and counts)
- Cats (presence-absence and counts)
- CCZ visits in the last 12 months (presence-absence)
- Use of rodenticides (presence-absence)

Hypothesis for each model

Sex model

- Opossums: opossums might predate a certain sex more commonly due to territorial behaviors, and/or cause females to avoid certain areas for risk of predation of the young.
- Ground coverage: pervious soil could be attracting more females due to the available soil for burrows.
- Available residues: areas with high amounts of resources might pool in more of one sex to defend them.
- Water bodies: water bodies are important pathways for Norway rat’s movement in the landscape. The presence of water bodies could be the entrance point of males displaced from their groups by a dominant individual.
- Presence of sewers: sewers could be the entrance point of males displaced from their groups by a dominant individual.
- CCZ visits: the visits, that usually end with application of rodenticides, could be culling one sex more than the other due to exploration behaviors.
- Rodenticides: rodenticides could be culling one sex more than the other due to exploration behaviors
- Cats and dogs: these animals act as predators, and could be culling one sex more than the other due to territorial and exploration behaviors

Maturity model

- Dogs and cats: these animals act as predators, and could be culling more young naïve individuals that are either exploring the environment, or have not learned to avoid predation.
- CCZ visits: the application of rodenticides could be affecting more one age group, as the poison is applied in the burrows and their direct vicinities.
- Rodenticide: the application of rodenticides could be affecting more one age group, as the poison is applied in the burrows and their direct vicinities.
- Ground coverage: areas with pervious soil will gather more adult individuals, as they build their burrows.
- Water bodies: water bodies could be causing an influx of younger individuals, arriving displaced from groups by a dominant male.
- Sewers: sewers could be causing an influx of younger individuals, arriving displaced from groups by a dominant male.
- Available residues: more adults could be concentrated in an area with abundant resources to defend it from outsiders.
- Opossums: opossums could be predating young individuals preferentially as they are smaller and might not be prepared to fend off a predator.

Wounds model

- Cats and dogs: these animals are predators, and predation can be seen as a stressor that could induce more agonistic interactions between members of a colony.
- CCZ visits: the use of rodenticides could be a modulator of wounds by removing individuals from the population and alleviating competition.
- Rodenticide: the use of rodenticides could be a modulator of wounds by removing individuals from the population and alleviating competition.
- Ground coverage: areas with pervious soil will have animals with more wounds due to territorial defense.
- Category of water body: animals captured near water bodies are likely to be wounded due to escaping or migrating away from a dominant male.
- Sewers: animals captured near sewers are likely to be wounded due to escaping or migrating away from a dominant male.
- Available residues and materials: areas rich in resources will be populated by animals with wounds caused by territory defense.
- Opossums: opossums could both be predating selectively an age class and deflating the rate of agonistic encounters, as well as being responsible by some of the wounds themselves.

SMI model

- Cats and dogs: these animals are predators, and predation can be seen as a stressor that could affect the population’s body condition.
- CCZ visits: animals exposed to poison might not die, but have an aggravated body condition.
- Rodenticide: animals exposed to poison might not die, but have an aggravated body condition.
- Ground coverage: areas with pervious soil might be occupied selectively by larger animals that displace smaller competitors.
- Category of water body: animals captured near water bodies are likely to be smaller, migrating away from a dominant male.
- Sewers: animals captured near sewers are likely to be smaller, migrating away from a dominant male.
- Available residues and materials: areas rich in resources will be populated by larger dominant animals.
- *Capillaria hepatica*: a parasite naturally worsens its host’s body condition.

Section 2 – Eveness tests for communities

Code:

#sex

dmsub %>% select (rat_id,area,sex_bin) %>% group_by (area, sex_bin) %>% summarise(n=n()) %>% mutate(freq= n/sum(n)) %>% kable

summary(glm(sex_bin ~ area, family= binomial, data=dmsub))

#weight

dmsub %>% select (rat_id,area,weight) %>% group_by (area) %>% summarise(xweight=mean(weight))%>% kable

summary(lm(weight ~ area, data=dmsub))

#maturity

dmsub %>% select (rat_id,area,maturity_bin) %>% group_by (area, maturity_bin) %>% summarise(n=n()) %>% mutate(freq= n/sum(n)) %>% kable

summary(glm(maturity_bin ~ area, family= binomial, data=dmsub))

Areas x Sex (Summary)

| Area | Sex | N | Frequency |
| --- | --- | --- | --- |
| AC | Female | 21 | 0.5676 |
| AC | Male | 16 | 0.4324 |
| MR | Female | 13 | 0.619 |
| MR | Male | 8 | 0.381 |
| NC | Female | 20 | 0.5263 |
| NC | Male | 18 | 0.4737 |
| RS | Female | 8 | 0.44 |
| RS | Male | 10 | 0.56 |

Deviance Residuals:

Min 1Q Median 3Q Max

-1.2735 -1.0643 -0.9794 1.2225 1.3893

Coefficients:

Estimate Std. Error z value Pr(>|z|)

(Intercept) -0.2719 0.3318 -0.819 0.413

areaMR -0.2136 0.5586 -0.382 0.702

areaNC 0.1666 0.4644 0.359 0.720

areaRS 0.4951 0.5789 0.855 0.392

(Dispersion parameter for binomial family taken to be 1)

Null deviance: 157.16 on 113 degrees of freedom

Residual deviance: 155.83 on 110 degrees of freedom

AIC: 163.83

Number of Fisher Scoring iterations: 4

Area x Weight

| Area | Weight |
| --- | --- |
| AC | 269.459 |
| MR | 225.69 |
| NC | 285.447 |
| RS | 261.111 |

Residuals:

Min 1Q Median 3Q Max

-255.447 -65.945 0.541 74.310 174.553

Coefficients:

Estimate Std. Error t value Pr(>|t|)

(Intercept) 269.459 15.528 17.354 <2e-16 ***

areaMR -43.769 25.805 -1.696 0.0927 .

areaNC 15.988 21.814 0.733 0.4652

areaRS -8.348 27.142 -0.308 0.7590

Residual standard error: 94.45 on 110 degrees of freedom

Multiple R-squared: 0.04786, Adjusted R-squared: 0.02189

F-statistic: 1.843 on 3 and 110 DF, p-value: 0.1436

Area x Maturity

| Area | Maturity | N | Frequency |
| --- | --- | --- | --- |
| AC | Juvenile | 8 | 0.2162 |
| AC | Adult | 29 | 0.7838 |
| MR | Juvenile | 11 | 0.5238 |
| MR | Adult | 10 | 0.4762 |
| NC | Juvenile | 6 | 0.1579 |
| NC | Adult | 32 | 0.8421 |
| RS | Juvenile | 5 | 0.2778 |
| RS | Adult | 13 | 0.7222 |

Deviance Residuals:

Min 1Q Median 3Q Max

-1.9214 -1.1372 0.5863 0.6980 1.2181

Coefficients:

Estimate Std. Error z value Pr(>|z|)

(Intercept) 1.2879 0.3994 3.225 0.00126 **

areaMR -1.3832 0.5919 -2.337 0.01946 *

areaNC 0.3861 0.5978 0.646 0.51836

areaRS -0.3323 0.6606 -0.503 0.61490

(Dispersion parameter for binomial family taken to be 1)

Null deviance: 131.40 on 113 degrees of freedom

Residual deviance: 122.12 on 110 degrees of freedom

AIC: 130.12

Number of Fisher Scoring iterations: 4
